# Effect of low doses of estradiol and tamoxifen on breast cancer cell karyotypes

**DOI:** 10.1530/ERC-16-0078

**Published:** 2016-08-01

**Authors:** Milena Rondón-Lagos, Nelson Rangel, Ludovica Verdun Di Cantogno, Laura Annaratone, Isabella Castellano, Rosalia Russo, Tilde Manetta, Caterina Marchiò, Anna Sapino

**Affiliations:** 1 Department of Medical SciencesUniversity of Turin, Turin, Italy; 2 Natural and Mathematical Sciences FacultyUniversidad del Rosario, Bogotá, Colombia; 3 Pathology DivisionAzienda Ospedaliera Città della Salute e della Scienza di Torino, Turin, Italy; 4 Department of Public Health and PediatricsUniversity of Turin, Turin, Italy; 5 Candiolo Cancer InstituteFPO-IRCCS, Candiolo, Italy

**Keywords:** breast cancer cells, estradiol, tamoxifen, chromosomal abnormalities, chromosomal instability

## Abstract

Evidence supports a role of 17&-estradiol (E_2_) in carcinogenesis and the large majority of breast carcinomas are dependent on estrogen. The anti-estrogen tamoxifen (TAM) is widely used for both treatment and prevention of breast cancer; however, it is also carcinogenic in human uterus and rat liver, highlighting the profound complexity of its actions. The nature of E_2_- or TAM-induced chromosomal damage has been explored using relatively high concentrations of these agents, and only some numerical aberrations and chromosomal breaks have been analyzed. This study aimed to determine the effects of low doses of E_2_ and TAM (10^&8 ^mol L^&1^ and 10^&6 ^mol L^&1^ respectively) on karyotypes of MCF7, T47D, BT474, and SKBR3 breast cancer cells by comparing the results of conventional karyotyping and multi-FISH painting with cell proliferation. Estrogen receptor (ER)-positive (+) cells showed an increase in cell proliferation after E_2_ treatment (MCF7, T47D, and BT474) and a decrease after TAM treatment (MCF7 and T47D), whereas in ER& cells (SKBR3), no alterations in cell proliferation were observed, except for a small increase at 96 h. Karyotypes of both ER+ and ER& breast cancer cells increased in complexity after treatments with E_2_ and TAM leading to specific chromosomal abnormalities, some of which were consistent throughout the treatment duration. This genotoxic effect was higher in HER2+ cells. The ER&/HER2+ SKBR3 cells were found to be sensitive to TAM, exhibiting an increase in chromosomal aberrations. These *in vitro* results provide insights into the potential role of low doses of E_2_ and TAM in inducing chromosomal rearrangements in breast cancer cells.

## Introduction

17&-estradiol (E_2_) is the main estrogenic hormone that through the estrogen receptors (ER) acts on the mammary gland regulating a wide variety of biological processes including differentiation, cell proliferation, and development at puberty and during sexual maturity. E_2_ may be procancerogenic by inducing (i) ER-mediated cell proliferation, (ii) gene mutation through a cytochrome P450-mediated metabolic activation, and (iii) aneuploidy (Russo & Russo 2006), through overexpression of Aurora-A (Aur-A), a centrosome kinase, and centrosome amplification (Li *et al*. 2004). In addition, in both ER+ and ER& breast cancer cells, E_2_ may induce chromatin structural changes through the estrogen-related receptors (ERR) (Hu *et al*. 2008). Although high levels of E_2_ are implicated in breast cancer in postmenopausal women (Bernstein & Ross 1993), constant low E_2_ concentrations, in the range of picograms, are sufficient to increase breast cancer risk in premenopausal women (Chetrite *et al*. 2000).

Tamoxifen (TAM) is a non-steroidal anti-estrogen with partial agonistic activity, extensively used in the treatment of ER&-positive breast cancer. Response to TAM is frequently of limited duration due to the development of resistance (Pearce & Jordan 2004, International Breast Cancer Study *et al*. 2006). Although ER& positivity is a well-established predictor of response to TAM and ER&-negative patients are considered nonresponders, it is known that 5–10% of  ER&-negative tumors do benefit from adjuvant TAM treatment (McGuire 1975, Early Breast Cancer Trialists’ Collaborative Group 1992, 1998, Early Breast Cancer Trialists’ Collaborative Group *et al*. 2011, Gruvberger-Saal *et al*. 2007).

Paradoxically, it has been reported that TAM possesses a high mutagenic potential causing chromosome ruptures in animal models (Mizutani *et al*. 2004). However, data on type and frequency of chromosome abnormalities induced by TAM are scant (Mizutani *et al*. 2004). In particular, cytogenetic studies about the effects of low doses of TAM, as it is suggested for treatment of pre-invasive low-grade breast lesions (e.g., low-grade ductal carcinomas *in situ* or lobular intraepithelial neoplasia), are limited (Kedia-Mokashi *et al*. 2010). The nature of E_2_- or TAM-induced chromosomal damage has been explored using relatively high concentrations of these agents, and only some numerical aberrations and chromosomal breaks have been analyzed (Tsutsui & Barrett 1997, Mizutani *et al*. 2004, Quick *et al*. 2008, Kedia-Mokashi *et al*. 2010).

The aim of this study was to determine the effects of low doses of E_2_ and TAM on chromosomal rearrangements by comparing the results of conventional karyotyping and multicolor fluorescence *in situ* hybridization (M-FISH) painting with cell proliferation activity of human breast cancer cells with differential expression of ER and HER2.

## Materials and methods

### Cell lines

The human breast cancer cell lines MCF7 and T47D (ER+/progesterone receptor (PR)+/HER2&), BT474 (ER+/PR+/HER2+), and SKBR3 (ER&/PR&/HER2+) were obtained from the American Type Culture Collection (ATCC) in March 2010. Cell lines were expanded and stocked at &80°C and cells obtained from these stocks were thawed and used for the experiments. At the end of experiments, short tandem repeat (STR) profiles were performed to confirm the authentication of the cell lines used. All experiments were carried out in each cell line at passages (P) below 30.

MCF7 (P19), T47D (P20), and SKBR3 (P16) were cultured in RPMI-1640 medium (Sigma), whereas BT474 (P18) was cultured in DMEM medium (Sigma). All culture media were supplemented with 10% fetal bovine serum (FBS) (Sigma), antibiotic–antimycotic solution (1X) (Sigma), and l-glutamine (2 mM) (Invitrogen GmbH). Cells growing in 75 cm^2^ flasks were maintained at 37°C and 5% CO_2_. The absence of contamination with mycoplasma was demonstrated by PCR assay.

### E_2_ and TAM treatment

In order to remove endogenous serum steroids and exclude the weak estrogen agonistic activity of phenol red (Berthois *et al*. 1986), 48 h before the addition of E_2_ (E2758; Sigma) and TAM (T5648; Sigma) cells were washed with 5 mL phosphate-buffered saline (PBS) and then switched to phenol red-free RPMI-1640 (Sigma) containing 10% charcoal-stripped FBS (Sigma). E_2_ and TAM were dissolved in absolute ethanol and diluted in the media at 10^&8 ^mol L^&1^ and 10^&6 ^mol L^&1^, respectively, and then added to the culture medium at 24, 48, and 96 h. These concentrations have been demonstrated to be the lowest to induce an effect on the architecture of the cytoskeleton in breast cancer cells *in vitro* (Sapino *et al*. 1986).

Cells without treatment at 24 h (T24 h) and at 96 h (T96 h) were used as controls.

### Proliferation assay

Cells were seeded at a density of 2.5–5 × 10^3^ cells per 100 &L of phenol red-free medium in a 96 multi-well plate and after 24 h were treated with E_2_ and TAM for 24, 48, and 96 h. At the end of each treatment, cell proliferation was assessed using the cell proliferation ELISA kit, BrdU (Roche Diagnostics Deutschland GmbH). Measurement of absorbance was performed by using a MultiSkan Bichromatic reader (Labsystems, Midland, Canada) against a background control as blank. Each treatment was performed in 24 replicates and expressed as means ± standard deviation (s.d.).

### Metaphase spreads and G-banding

To determine whether E_2_ and TAM treatment resulted in the induction of chromosomal abnormalities, we performed conventional and molecular cytogenetic analysis in parallel with the evaluation of cell proliferation. Metaphases were obtained by using standardized harvesting protocols in order to perform conventional and molecular cytogenetic analysis (multi-FISH and FISH). Briefly, colcemid solution (0.03 &g/mL) (Sigma) was added to cultures 2.5 h before cell harvesting; cells were then treated with hypotonic solution, fixed three times with Carnoy’s fixative (3:1 methanol to acetic acid), and spread on glass. For analysis of chromosomal alterations, the slides were banded with G-banding. Glass slides were baked at 70°C for 24 h, incubated in HCl, and placed in 2xSSC buffer before treatment with Wright’s stain. Metaphase image acquisition and subsequent karyotyping were performed using a Nikon microscope with the cytogenetic software CytoVision System (Applied Imaging, Santa Clara, CA, USA). According to the International System of Cytogenetic Nomenclature (Shaffer *et al*. 2013) “The general rule in tumor cytogenetics is that only the clonal chromosomal abnormalities should be reported”, whereas a minimal number of metaphases to be analyzed is not indicated. In this respect, we indicated only those alterations present in at least two metaphases, which is indicative of clonal chromosomal alterations (Shaffer *et al*. 2013). Based on these premises, we systematically analyzed 100 metaphases in order to establish the frequency of ploidy after treatments, by counting the number of chromosomes. As a second step, out of these metaphases, only those with good morphology and proper separation of chromosomes were analyzed by M-FISH and G-banding (between 11 and 26). Chromosome aberrations were described according to the International System of Human Cytogenetic Nomenclature (ISCN 2013) (Shaffer *et al*. 2013).

### Multi-FISH (M-FISH)

M-FISH was performed with the aim of identifying complex chromosomal rearrangements. The probe cocktail containing 24 differentially labeled chromosome-specific painting probes (24xCyte kit MetaSystems, Altlussheim, Germany) was used according to the protocol recommended by Human Multicolor FISH kit (MetaSystems, Altlussheim, Germany). Briefly, the slides were incubated at 70°C in saline solution (2xSSC), denatured in NaOH, dehydrated in ethanol series, air-dried, covered with 10 &L of probe cocktail (denatured), and hybridized for 2 days at 37°C. Slides were then washed with post-hybridization buffers, dehydrated in ethanol series, and counterstained with 10 &L of DAPI/antifade. Signal detection and subsequent metaphase analysis were done using the Metafer system and Metasytems’ ISIS software (software for spectral karyotypes) (Carl Zeiss, Metasystems, GmbH, Germany) (Rondon-Lagos *et al*. 2014*a*,*b*).

### Immunohistochemistry (IHC)

Immunohistochemistry for ER and PR was carried out on MCF7, T47D, BT474, and SKBR3 cells at baseline and treated with E_2_ (10^&8 ^mol L^&1^) and TAM (10^&6 ^mol L^&1^) for 24, 48, and 96 h. At each time point, cells were harvested, formalin-fixed, and paraffin-embedded according to standard procedures. Sections of the representative cell block were cut at 3 &m and mounted on electrostatically charged slides. Immunohistochemistry was performed using an automated immunostainer (Ventana BenchMark XT AutoStainer; Ventana Medical Systems, Tucson, AZ, USA) with antibodies against ER (Clone SP1, prediluted, Ventana) and PR (Clone 1A6, 1:50 diluted; Leica Biosystems). Positive and negative controls were included for each immunohistochemical run. IHC slides were scanned by using the Aperio system (ScanScope CS System, Vista, CA, USA) for automated counting. To ensure the reliability of the automatic assessment, stainings were reviewed by two pathologists (A S and C M).

### Data analysis

The profile of numeric and structural chromosomal changes observed after treatments was determined in comparison with the control. Student’s *t*-test was performed to compare cell proliferation of treated cell lines with untreated cell lines. Fisher’s exact test was applied to compare conventional and molecular cytogenetic results from treated cell lines with the results from control cell lines (differences in single chromosomal alterations between control and treated cells). In addition, Pearson’s *&*^2^ test was used to investigate a possible association between occurrence of specific chromosomal aberrations at each time point and effect on proliferation. The coefficient of variation, CV (=100 × standard deviation/mean), was used to calculate the variability in the frequency of new chromosomal alterations, observed after E_2_ and TAM treatments (24, 48, and 96 h). *P* values <0.05 were considered as statistically significant. All statistical analyses were performed using the SPSS v.20 program.

## Results

### General effects on chromosomes induced by low doses of E_2_ and TAM

Control cells harbored the same alterations previously reported (Rondon-Lagos *et al*. 2014*a*,*b*). Both E_2_ and TAM treatments rapidly induced *de novo* chromosomal alterations.

**Figure 1:**
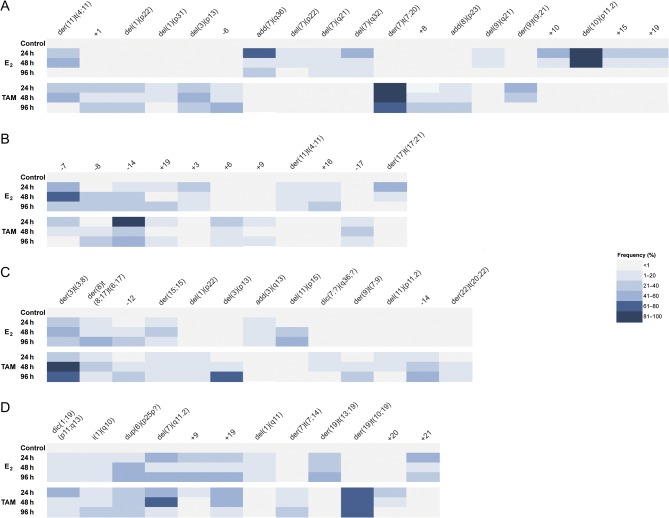
Frequency of chromosomal alterations observed *de novo* after E_2_ and TAM treatments. The frequency of each chromosomal alteration is indicated along the treatments (24, 48, and 96 h) using a color code for each category. (A) MCF7 cells. (B) T47D cells. (C) BT474 cells. (D) SKBR3 cells. A full colour version of this figure is available at http://dx.doi.org/10.1530/ERC-16-0078.

The frequency of new chromosomal alterations changed along E_2_ and TAM treatments for all cell lines, and while the frequency of some chromosomal abnormalities remained constant along treatments, other increased or decreased (CV range: 3–96%) (Fig. 1 and Supplementary Table 1, see section on supplementary data given at the end of this article). This variability is not surprising, considering that genetic diversification, clonal expansion, and clonal selection are events widely reported in cancer and also associated with therapeutic interventions (Greaves & Maley 2012).

**Figure 2:**
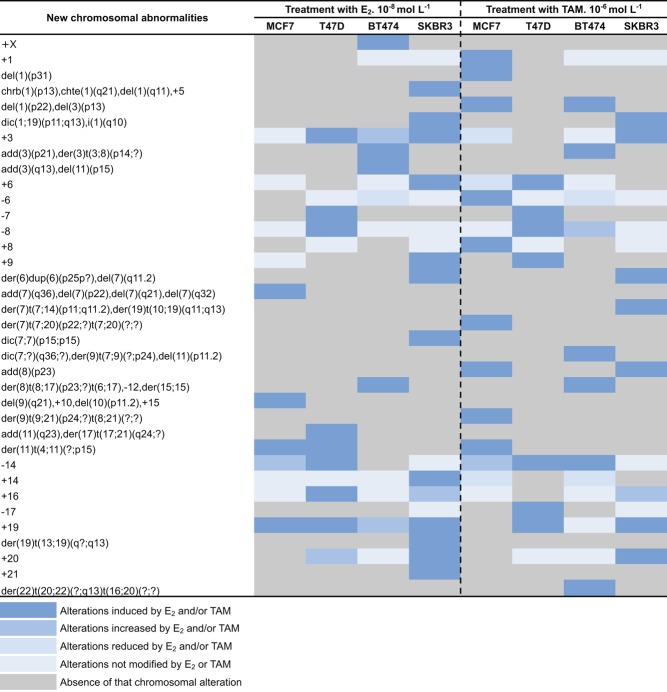
Clonal chromosomal abnormalities induced by E_2_ and TAM in four breast cancer cell lines at each treatment time point. The presence of a given chromosomal alteration after E_2_ and/or TAM treatment in one or more cell lines is color coded according to the legend at the bottom. A full colour version of this figure is available at http://dx.doi.org/10.1530/ERC-16-0078.

**Figure 3:**
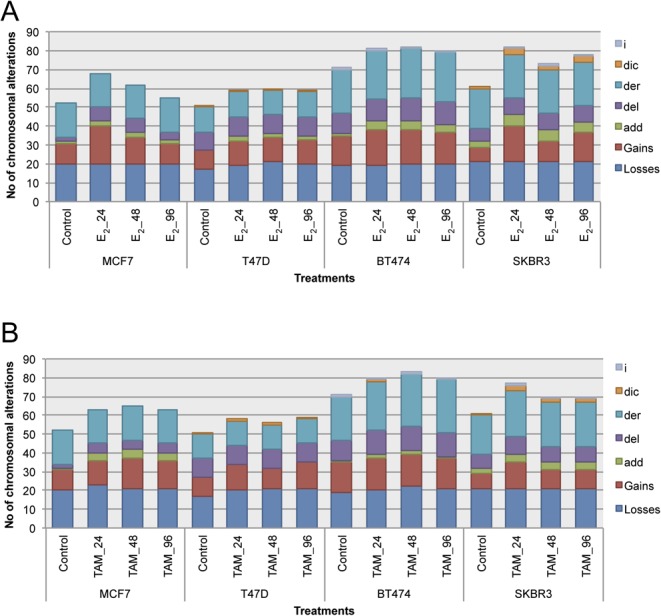
Total number of chromosomal aberrations induced after E_2_ (A) and TAM (B) treatment at 24, 48, and 96 h in MCF7, T47D, BT474, and SKBR3 cell lines. Numerical chromosomal alterations: gains and losses. Structural chromosomal alterations: add, additional material of unknown origin; del, deletion; der, derivative chromosome; dic, dicentric chromosome;  i, isochromosome. A full colour version of this figure is available at http://dx.doi.org/10.1530/ERC-16-0078.

More in detail, compared with control cells (T24 h and T96 h without treatment), low doses of E_2_ increased the chromosome ploidy in all cell lines (Table 1A), whereas TAM was effective on ploidy only in HER2+ cell lines (Table 1B). Some of the alterations were observed in more than one cell line and were induced by both E_2_ and TAM (Fig. 2 and Supplementary Table 2). In Fig. 3, the chromosomal aberrations induced or increased after E_2_ or TAM treatments as compared with control cells are represented. Low doses of E_2_ produced numerical alterations represented mainly by gain of whole chromosomes in all cell lines. Low doses of both E_2_ and TAM induced *de novo* structural aberrations such as isochromosomes (i) in BT474 and SKBR3 cells and dicentric (dic) chromosomes in T47D and BT474 cells. Both treatments increased derivative (der) chromosomes in HER2+ cells only, whereas additional material of unknown origin (add) was a *de novo* observation only in T47D after E_2_ treatment.

**Table 1:** Percentage of cells with polyploidy in MCF7, T47D, BT474, and SKBR3 cell lines. (A) Control and E_2_ treated. (B) Control and TAM treated. A hundred metaphases were analyzed for both control and for each of the treatments with E_2_ and TAM.

**Treatments**	**MCF7**		**T47D**		**BT474**		**SKBR3**	
	4n	>4n	3n	>3n	4n	>4n	4n	>4n
A								
Control	98	2	96	4	100	0	81	19
E2. 24 h	85	15	87	13	88	12	63	37
E2. 48 h	80	20	78	22	77	23	52	48
E2. 96 h	61	39	67	33	70	30	50	50
B								
Control	98	2	96	4	100	0	81	19
TAM. 24 h	97	3	85	15	94	6	24	76
TAM. 48 h	99	1	98	2	98	2	24	76
TAM. 96 h	99	1	100	0	84	16	30	70

Many of the altered chromosomal regions in the cell lines analyzed contain important genes involved in breast cancerogenesis including *BCAR3* (1p22), *CENPF* (1q41), *ENAH* (1q42), and *AKT3* (1q44) associated with aneuploidy, chromosomal instability, and anti-estrogen resistance (Nakatani *et al*. 1999, Di Modugno *et al*. 2006, O’Brien *et al*. 2007); *FHIT*, *FOXP1*, and *LRIG1* on 3p14 correlated with chromosomal instability and anti-estrogen resistance (Campiglio *et al*. 1999, Banham *et al*. 2001, Ljuslinder *et al*. 2005); *AKAP9*(7q21), *DMTF1*(7q21), and *HIPK2* (7q32) involved in the assembly of protein kinases to the centrosome and in growth arrest (Edwards & Scott 2000, Sreeramaneni *et al*. 2005, Pierantoni *et al*. 2007); *E2F1* (20q11.22) and *MAPRE1* (20q11.1-11.23) involved in the regulation of the mitotic cell division process, regulation of microtubule dynamic instability, and in cell cycle control (Stender *et al*. 2007), among others (Table 2).

**Table 2:** Selected breast cancer oncogenes and tumor suppressor genes present in the chromosomal regions affected by chromosomal abnormalities in MCF7, T47D, BT474, and SKBR3 cell lines following treatment with E_2_ and TAM for 24, 48, and 96 h.

Chromosomal region		Cell line					
	Genes	MCF7	T47D	BT474	SKBR3	Function	References
1p13.3	*CSF1*				X	Cell proliferation	www.ncbi.nlm.nih.gov
1p22	*BCL10*	X		X		Oncogene, apoptosis	Lin (2009)
1p22	*BCAR3*	X		X		Cell proliferation, resistance in breast cancer cell lines	Nakatani *et al*. (1999), Di Modugno *et al*. (2006), O’Brien *et al*. (2007)
1p32p31	*JUN*				X	Oncogen	www.ncbi.nlm.nih.gov
1p36.21	*PRDM2*				X	Tumor suppressor gene, binds to ER. Transcriptional regulation, E_2_ effector action	www.ncbi.nlm.nih.gov
1q11	*MUC1*				X	Cell physiology and pathology, up-regulated in breast cancer	Zaretsky *et al*. (2006)
1q21.1	*CA14*				X	Basic cellular metabolism; breast cancer	Orsetti *et al*. (2006), Beroukhim *et al*. (2010), Bignell *et al*. (2010)
1q21.3	*PIP5K1A*				X	Cell proliferation, breast cancer	Orsetti *et al*. (2006), Beroukhim *et al*. (2010), Bignell *et al*. (2010)
1q25.2-q25.3	*COX2*				X	Inflammation and mitogenesis	www.ncbi.nlm.nih.gov
1q32	*KISS*				X	Cell motility, oncogene	Orsetti *et al*. (2006), Bignell *et al*. (2010), Beroukhim *et al*. (2010)
1q31	*PTGS2*				X	Inflammation, tumorigenesis	Dossus *et al*. (2010)
1q41	*CENPF*				X	Kinetochore assembly	Nakatani *et al*. (1999), Di Modugno *et al*. (2006), O’Brien *et al*. (2007)
1q42.12	*ENAH*				X	Cell shape and movement	Nakatani *et al*. (1999), Di Modugno *et al*. (2006), O’Brien *et al*. (2007)
1q44	*AKT3*				X	Proliferation, cell survival, and tumorigenesis	Nakatani *et al*. (1999), Di Modugno *et al*. (2006), O’Brien *et al*. (2007)
3p14	*FHIT*			X		Tumor suppressor gene; resistance to tamoxifen in MCF7 cells	Campiglio *et al*. (1999)
3p14	*FOXP1*			X		Tumor suppressor gene, multiple types of cancers	Banham *et al*. (2001)
3p14	*LRIG1*			X		Suppressor of receptor tyrosine kinases, breast cancer	Ljuslinder *et al*. (2005)
6p25	*TFAP2A*				X	Tumor supressor gene, breast cancer	Scibetta *et al*. (2010)
6p25	*DUSP22*				X	Signaling pathway, breast cancer	Curtis *et al*. (2012)
7p22	*GPR30*	X				G protein-coupled receptor 30, drug resistance	Wang *et al*. (2010)
7p22	*SDK1*	X				Cell adhesion protein, breast cancer	Curtis *et al*. (2012)
7q11.2	*LIMK1*				X	Organization of actin cytoskeleton	Laskowska *et al*. (2010)
7q11.2	*HSPB1*				X	Oncogenesis and resistance to various anti-cancer therapies	Laskowska *et al*. (2010)
7q11.2	*AUTS2*				X	Breast cancer	www.ncbi.nlm.nih.gov
7q21	*AKAP9*	X				Protein that assembles protein kinases on the centrosome	Edwards & Scott (2000)
7q21	*DMTF1*	X				Transcriptional activator promoting p53/TP53-dependent growth arrest.	Sreeramaneni *et al*. (2005)
7q32	*HIPK2*	X				Tumor supressor gene, breast cancer	Pierantoni *et al*. (2007)
7q36	*MNX1*	X				Transcription factor, breast cancer	Nik-Zainal *et al*. (2012)
7q36	*MLL3*	X				Transcriptional coactivation, breast cancer	Nik-Zainal *et al*. (2012)
8p22	*MTUS1*	X		X		Tumor suppressor gene, breast cancer	Rodrigues-Ferreira *et al*. (2009)
8p23	*CTSB*	X		X		Metabolism, angiogenesis, invasion, and metastasis in breast cancer	Rafn *et al*. (2012)
8p23	*CSMD1*	X		X		Tumor supressor gene, poor survival in breast cancer	Kamal *et al*. (2010), Curtis *et al*. (2012)
8p23	*DLC1*	X		X		Tumor suppressor gene, breast cancer	Popescu & Zimonjic (2002)
9p24	*JAK2*	X		X		Protein tyrosine kinase of the non-receptor type, breast cancer	Curtis *et al*. (2012)
9p24	*RLN2*	X		X		Development of mammary gland. Invasion in breast cancer	Radestock *et al*. (2008)
9p24	*KANK1*	X		X		Tumor supressor gene, breast cancer	Curtis *et al*. (2012)
9p24	*JMJD2C*	X		X		Demethylase, breast cancer	Curtis *et al*. (2012)
10p11.2	*ABI1*	X				Cell growth inhibitor, cancer progression, and prognosis	Cui *et al*. (2010)
11p15	*HRAS*	X	X			Signal transduction, tumor aggressiveness in breast cancer	Hae-Young Yong *et al*. (2011)
11p15	*CTSD*	X	X			Invasion and metastasis	www.ncbi.nlm.nih.gov
11p15	*CD151*	X	X			Signal transduction, breast cancer	Ivyna Bong *et al*. (2011)
11p15	*RRM1*	X	X			Tumor supressor gene, DNA repair	Kim *et al*. (2011)
11p15	*MMP26*	X	X			Migration and angiogenesis, breast cancer	Curtis *et al*. (2012)
11p15	*CDKN1C*	X	X			Negative regulator of cell cycle	www.ncbi.nlm.nih.gov
11q23	*ATM*		X			Tumor supressor gene, DNA repair	Roy *et al*. (2006)
11q23	*CRYAB*		X			Molecular chaperone function, metastasis in breast cancer	Chelouche-Lev *et al*. (2004)
11q23	*ETS1*		X			Transcripction factor, breast cancer	Lincoln & Bove (2005)
11q23	*CCND1*		X			Cell cycle G1/S transition, tumorigenesis in various carcinomas	Lundgren *et al*. (2008)
11q23	*PGR*		X			Signal transduction, breast cancer	www.ncbi.nlm.nih.gov
15q10	*BUB1B*			X		Mitotic spindle checkpoint, chromosomal instability in breast cancer	Scintu *et al*. (2007)
15q15	*THBS1*			X		Invasion, metastasis, angiogenesis	www.ncbi.nlm.nih.gov
15q26.3	*IGF1R*			X		Cell growth and survival control, breast cancer	Kang *et al*. (2014)
17q24	*BIRC5*		X			Apoptosis inhibition	www.ncbi.nlm.nih.gov
18q21.1	*SMAD4*	X				Transcription factor, breast cancer	Curtis *et al*. (2012), Nik-Zainal *et al*. (2012)
18q21.1	*BCL2*	X				Cell death, breast cancer	Curtis *et al*. (2012)
18q21.2	*DCC*	X				Apoptosis, breast cancer	Koren *et al*. (2003)
19q13	*ATF5*				X	Cell cycle progression, breast cancer	Al Sarraj *et al*. (2005), Watatani *et al*. (2007)
19q13	*LILRA6*				X	Receptor for class I MHC antigens, breast cancer	Curtis *et al*. (2012)
19q13	*CYP2A6*				X	Metabolism of pharmaceutical drugs, directly induced by estradiol	Higashi *et al*. (2007)
19q13	*TGFB1*				X	Cell division and death, imply in tamoxifen resistance in breast cancer	Achuthan *et al*. (2001), Popescu & Zimonjic (2002), Jansen *et al*. (2005), Ivanovic *et al*. (2006)
19q13	*CEACAM1*				X	Cell survival, differentiation, and growth, breast cancer	Luo *et al*. (1997), Riethdorf *et al*. (1997)
20q11.22	*E2F1*	X				Tumor suppressor gene	Stender *et al*. (2007)
20q13.1	*CDH4*	X				Cell adhesion proteins, breast cancer	Curtis *et al*. (2012)
20q13.1	*MMP9*	X				Metastasis and cancer cell invasion, breast cancer	Kousidou *et al*. (2004)
20q13.31	*AURKA*	X				Cell proliferation, breast cancer	Cox *et al*. (2006)
22q13	*ATF4*			X		Adaptation of cells to stress factors, multidrug resistant gene	Igarashi *et al*. (2007)
22q13	*SERHL2*			X		Breast cancer	Curtis *et al*. (2012)
22q13	*LARGE*			X		Breast cancer	Curtis *et al*. (2012)
22q13	*XRCC6*			X		Apoptosis induction, breast cancer	Nik-Zainal *et al*. (2012)

### Combined effects on cell proliferation and  chromosomal alterations

We then more specifically analyzed the chromosomal alterations in comparison with the effects on proliferation induced by E_2_ and TAM in each cell line. Although we did not observe a specific pattern of chromosomal aberrations that significantly correlated with either increased or decreased proliferation rates across cell lines, single aberrations significantly correlated with increase  or decrease of proliferation within each cell line, as detailed below.

**Figure 4:**
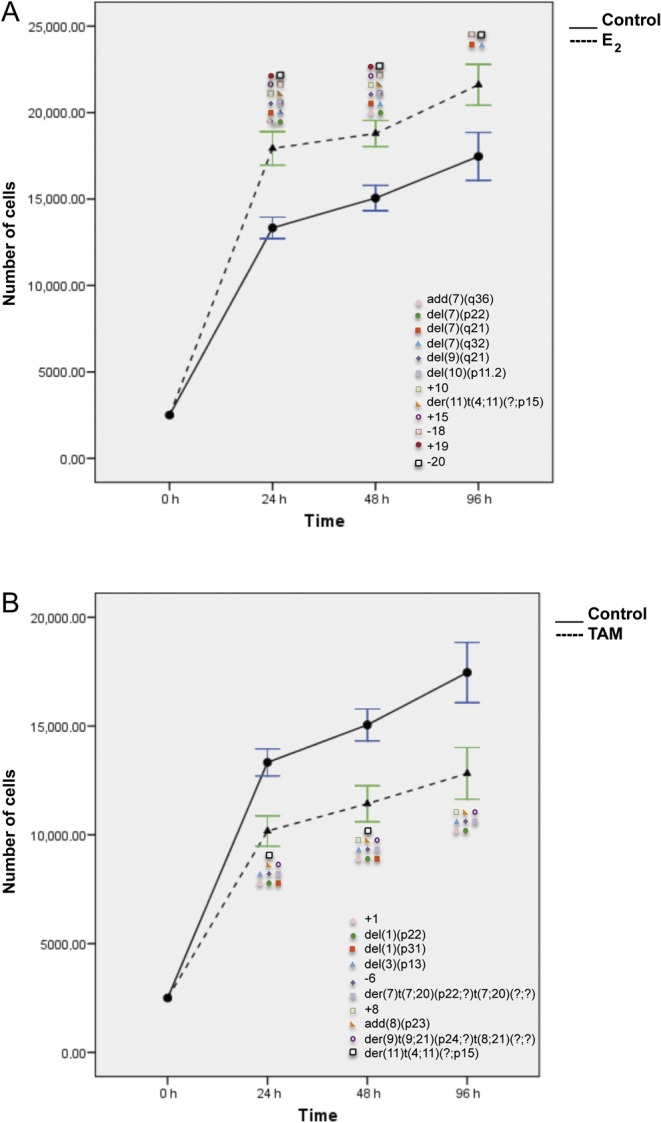
Effects of E_2_ (A) and TAM (B) treatment for 24, 48, and 96 h on proliferation and corresponding chromosomal alterations in MCF7 cells. Error bars represent mean standard deviation of 24 separate experiments. Chromosomal abnormalities induced at each treatment time point are indicated. A full colour version of this figure is available at http://dx.doi.org/10.1530/ERC-16-0078.

In MCF7 cell line, as expected, E_2_ treatment significantly stimulated cell proliferation (*P* < 0.0001, Student’s *t*-test; Fig. 4A) and induced more structural than numerical chromosomal alterations (*P *≤ 0.05, Fisher’s exact test; Fig. 2, Supplementary Tables 2, 3 and 4). However, only a statistically significant increase in nullisomy of chromosome 18 and 20 (*P *< 0.01) together with del(7)(q21) and del(7)(q32) was constantly observed at all treatment time points (Figs 2 and 4A, Supplementary Tables 3 and 4).

**Figure 5:**
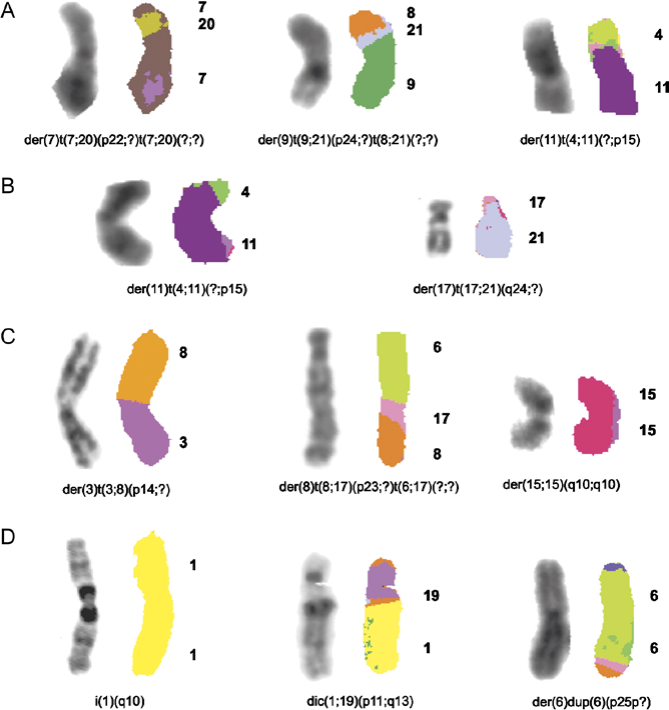
Representative images of chromosomal abnormalities observed throughout the treatment duration with either E_2_ or TAM. (A) MCF7 cells, (B) T47D cells, (C) BT474 cells, and (D) SKBR3 cells. Rearranged chromosomes are visualized by G-banding technique on the left and by M-FISH on the right. The chromosomes involved in the rearrangement are numbered on the right hand side of the chromosomes. A full colour version of this figure is available at http://dx.doi.org/10.1530/ERC-16-0078.

TAM treatment inhibited significantly MCF7 cell proliferation (*P* < 0.01) (Fig. 4B). Eleven chromosomes  (1, 2, 6, 7, 8, 10, 11, 17, 15, 19, and 20) varied in their copy number, but most of these alterations, except for +1 and &6, were observed only in one of the treatment time points and were considered as sporadic (Supplementary Table 3). As compared with control cells, six additional complex chromosomal aberrations, del(1)(p22), del(3)(p13), der(7)t(7;20)(p22;?)t(7;20)(?;?), add(8)(p23), der(9)t(9;21)(p24;?)t(8;21)(?;?), and der(11)t(4;11)(?;p15) (Figs 2, 4B, 5A and Supplementary Table 2), were identified and constantly present at each time point. In addition, der(11)t(4;11)(?;p15) was observed in both E_2_- and TAM-treated cells. An increase in the frequency of two pre-existing alterations del(7)(q11.2) and del(12)(p11.2) was also observed after both E_2_ and TAM treatment (Supplementary Table 4).

**Figure 6:**
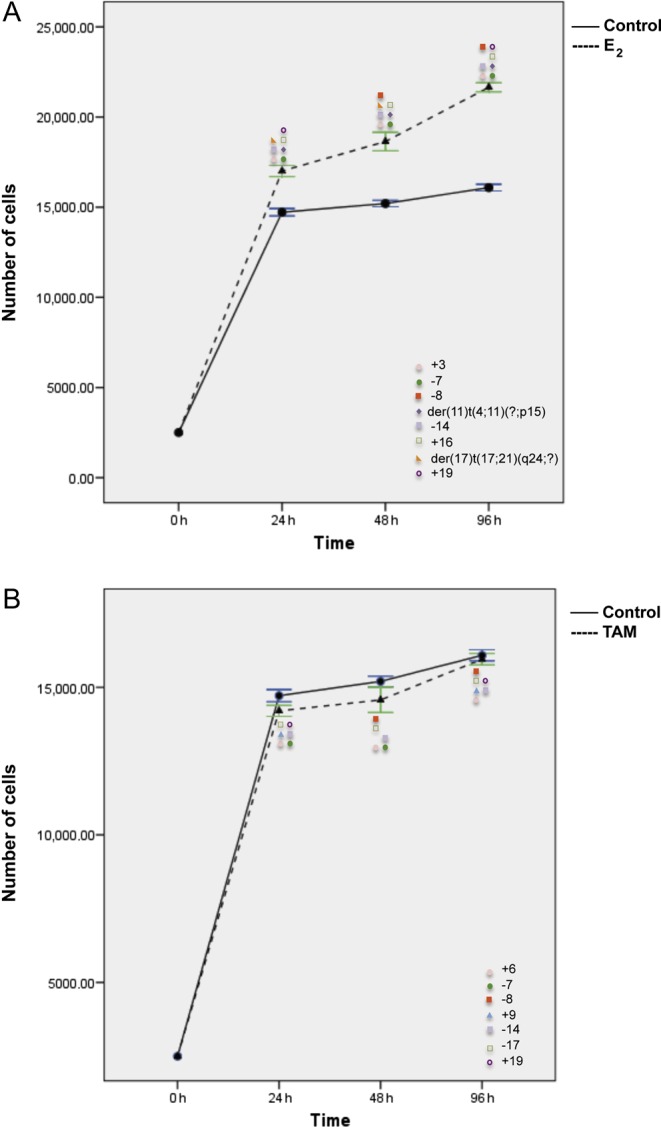
Effects of E_2_ (A) and TAM (B) treatment for 24, 48, and 96 h on proliferation and corresponding chromosomal alterations in T47D cells. Error bars represent mean standard deviation of 24 separate experiments. Chromosomal abnormalities induced at each treatment time point are indicated. A full colour version of this figure is available at http://dx.doi.org/10.1530/ERC-16-0078.

T47D cells responded to E_2_ treatment with the highest growth advantage at 96 h (Fig. 6A). This effect corresponded to a more complex karyotype of E_2_-stimulated cells than control cells with the following additional alterations, +3, &7, &8, der(11)t(4;11)(?;p15), &14, +16, and der(17)t(17;21)(q24;?) (*P* < 0.01), observed at least at two time points (Figs 2, 5B, 6A and Supplementary Table 2). In analogy to MCF7 cells, an increase in the frequency of some pre-existing numerical alterations was observed after both treatments in T47D cells (Supplementary Table 5).

The effect of TAM on cell growth inhibition was much lower than that observed in MCF7 cells and disappeared at 96 h (Fig. 6B). As compared with untreated controls, only three additional numerical alterations were constantly present (+6, &14, and &17) (*P* < 0.01, Fisher’s exact test) after TAM (Fig. 6B, Supplementary Tables 5 and 6). On the contrary, some chromosomal rearrangements present in the control cells could not be observed after E_2_ and TAM treatment (Supplementary Table 6). In T47D, both E_2_ and TAM induced loss of chromosomes 7, 8, and 14, whereas an additional chromosome 19 was induced by both treatments in T47D and SKBR3 cells.

**Figure 7:**
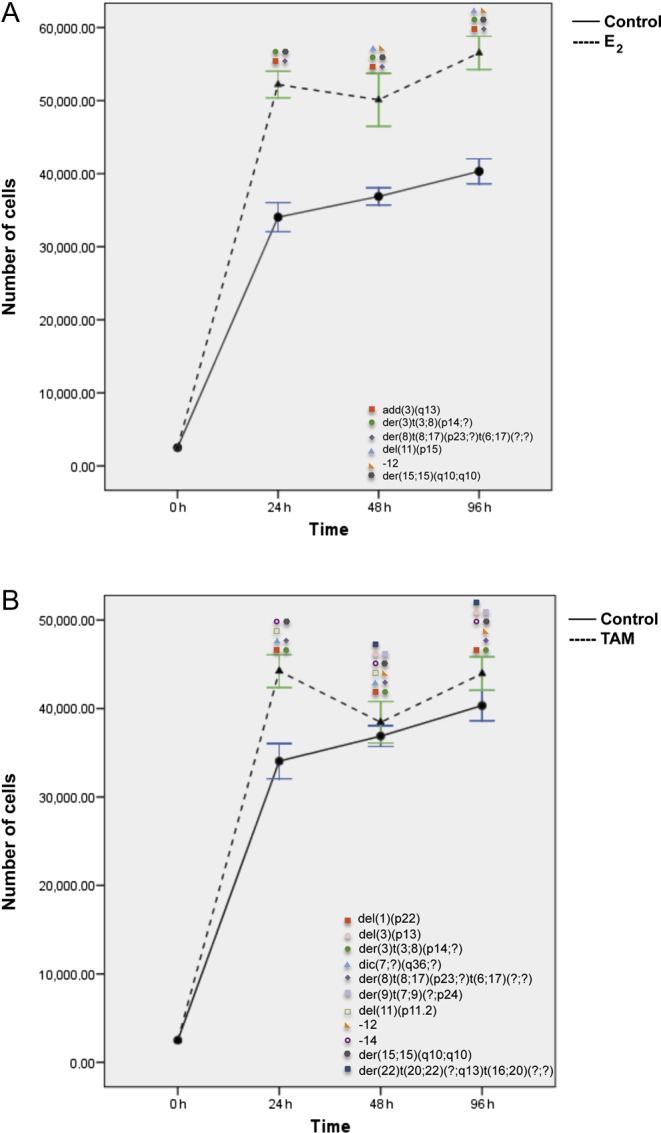
Effects of E_2_ (A) and TAM (B) treatment for 24, 48, and 96 h on proliferation and corresponding chromosomal alterations in BT474 cells. Error bars represent mean standard deviation of 24 separate experiments. Chromosomal abnormalities induced at each treatment time point are indicated. A full colour version of this figure is available at http://dx.doi.org/10.1530/ERC-16-0078.

In BT474 cells, both E_2_ and TAM treatments induced two peaks of proliferation at 24 and 96 h. G-banding and M-FISH analyses of both E_2_- and TAM-treated BT474 cells identified the same new chromosomal complex rearrangements der(3)t(3;8)(p14;?), der(8)t(8;17)(p23;?)t(6;17)(?;?), and der(15;15)(q10;q10) at each time point (Figs 2, 5C, 7 and Supplementary Table 2). Additional new rearrangements were observed after E_2_ (Fig. 7A, Supplementary Tables 7 and 8) or after TAM treatment (Fig. 7B) at least at two time points. An increase in the frequency of some preexisting chromosomal alterations (*P* ≤ 0.01) was also observed (Supplementary Tables 7 and 8).

**Figure 8:**
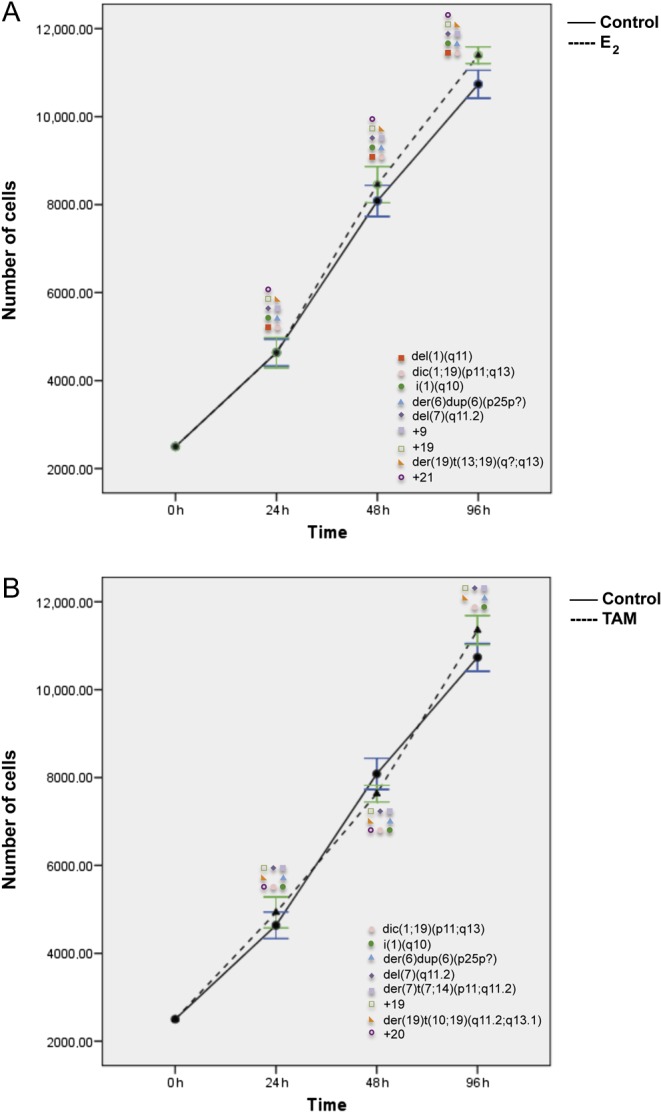
Effects of E_2_ (A) and TAM (B) treatment for 24, 48, and 96 h on proliferation and corresponding chromosomal alterations in SKBR3 cells. Error bars represent mean standard deviation of 24 separate experiments. Chromosomal abnormalities induced at each treatment time point are indicated. A full colour version of this figure is available at http://dx.doi.org/10.1530/ERC-16-0078.

Finally, in SKBR3 (ER&/HER2+), only 96 h of E_2_ and TAM treatment significantly increased cell proliferation (*P* < 0.006 and *P* < 0.024) (Fig. 8), as compared with controls. However, *de novo* chromosomal alterations were already observed after 24 h of treatment. SKBR3 control cells displayed a complex karyotype with a particularly high frequency of chromosome 1 aberrations. After 24 h of E_2_ and TAM treatment, the karyotype became even more complex with the appearance of new chromosome 1 abnormalities, such as for instance dic(1;19)(p11;q13) and i(1)(q10) (*P* < 0.05) (Figs 2, 5D, 8A, B and Supplementary Table 2). A statistically significant increase in the frequency of some pre-existing chromosomal abnormalities was observed in SKBR3 as well (Supplementary Tables 9 and 10).

### Expression of hormone receptors following treatment

IHC analysis showed that ER levels remained unchanged after E_2_ and TAM addition in MCF7, T47D, and SKBR3 cells, whereas in BT474 cells we observed an increase in both ER and PR expression after TAM treatment in parallel with an increase in proliferation (all time points; data not shown). These results support the hypothesis that TAM could play an estrogen agonist role in ER+/HER2+ cells (BT474), as it has been previously suggested (Pietras & Marquez-Garban 2007, Chang 2011, Kumar *et al*. 2011) and shown in other cell line models (Shou *et al*. 2004). In addition, increased PR expression in human breast cancers has been associated with TAM resistance (Cui *et al*. 2005).

E_2_ addition increased PR expression also in the other ER+ cell lines (MCF7 and T47D). In contrast, after TAM treatment, a reduced PR expression was observed in MCF7 and T47D cells (data not shown). This is in line with previous observations showing that when estradiol is acting, TAM is not able to increase the level of occupied estrogen receptors and it acts as an anti-estrogen by decreasing the high level of progesterone receptors previously induced by estradiol (Castellano-Diaz *et al*. 1989).

## Discussion

Short-term endocrine treatment has been proposed as an alternative to long-term neoadjuvant therapy to assess tumor response (Dowsett *et al*. 2007). In addition, low doses of TAM have been proposed for chemoprevention in women at high risk of developing breast cancer (Lazzeroni *et al*. 2012). Hypersensitivity to low levels of estrogen has been suggested as a potential mechanism of endocrine therapy resistance (Johnston & Dowsett 2003). In addition, residual amounts of estrogen may still be present after treatment with aromatase inhibitors, which function by reducing estrogen biosynthesis (Dowsett 1999). E_2_ binding to tubulin may induce a cell cycle arrest in G2/M and generate chromosomal instability (Sato *et al*. 1992, Sattler *et al*. 2003, Azuma *et al*. 2009, Lee *et al*. 2015).

In this study, we observed that low doses of both E_2_ and TAM were able to induce structural chromosomal aberrations (deletions, isochromosomes, translocations, and dicentric chromosomes) in both ER+ and ER& breast cancer cells.

Dicentric chromosomes, which contain two functional centromeres, can lead to extensive chromosomal rearrangements, including translocations with other chromosomes (Gascoigne & Cheeseman 2013). Chromosomal translocations, a frequent event observed after E_2_ and TAM treatment, may lead to the production of tumor-specific fusion proteins, which are often transcription factors (Rabbitts 1994). For example, der(11)t(4;11)(?;p15) was observed in both E_2_- and TAM-treated MCF7 cells and in E_2_-treated T47D. Several genes are located in the imprinted gene domain of 11p15.5, an important tumor-suppressor gene region (Hu *et al*. 1997).

While some complex chromosomal alterations were consistent throughout the treatments, other disappeared. The above could be related with the instability of such alterations. After treatment, unstable chromosomal alterations could be randomly fused to form more complex chromosomal rearrangements including translocations, dicentric chromosomes, and duplications (Shen 2013, Zhang *et al*. 2013). Another possible explanation, which can be strictly connected to the previous, is the possibility of clonal selection of the fittest clone (Heng *et al*. 2006, Liu *et al*. 2014, Dayal *et al*. 2015).

When chromosomal alterations were analyzed with respect to proliferation, some specific patterns within each cell line were observed. For instance, T47D cells showed a poorer response to TAM compared with MCF7 cells and mainly displayed numerical chromosomal alterations following treatment. The ER+/HER2+ BT474 cells showed the highest increase in cell proliferation after 24 h of treatment with both E_2_ and TAM compared with control cells. Cell growth increase after TAM treatment may indicate an estrogen agonist activity, possibly enhanced by the co-expression of ER and HER2 (Pietras & Marquez-Garban 2007, Chang 2011, Kumar *et al*. 2011). Indeed, the cross talk between ER pathways and growth factor receptor pathways (EGFR, IGF-1, and HER2) has been involved in cell proliferation, survival, and resistance to endocrine therapy (TAM) in breast cancer (Yager & Davidson 2006, Pietras & Marquez-Garban 2007, Chang 2011). However, in our study, after 48 h of TAM treatment cell proliferation decreased and increased again at 96 h. This decrease/increase may be explained through a clonal selection, with survival of those cells that acquired chromosomal abnormalities fostering proliferative and survival advantages.

As expected, our results confirm that the induction and inhibition of cell proliferation by E_2_ and TAM, respectively, is dependent on the presence of ER. However, in the  ER&/HER2+ SKBR3 cells, these agents induced a high frequency of chromosomal abnormalities and a small increase in proliferative activity at 96 h of treatment. Both effects may be due to the presence of the G protein-coupled receptor 30 (GPCR30), an estrogen transmembrane receptor, which modulates both rapid non-genomic and genomic transcriptional events of estrogen (Thomas *et al*. 2005,  Chen & Russo 2009, Li *et al*. 2010, Cheng *et al*. 2011). On the other hand, E_2_ may induce chromatin structural changes in both ER+ and ER& breast cancer cells through ERR (Hu *et al*. 2008). The ability of estrogens and its metabolites (catechol estrogens) to induce mutations in cancer cells has been demonstrated both *in vivo* and *in vitro* (Liehr 2000, Yager 2015), being observed that estrogens induce overexpression of the *Aurora A* and *B* genes (Li *et al*. 2004), cause genomic instability (Barrett *et al*. 1981, Tsutsui & Barrett 1997, Ahmad *et al*. 2000, Jeruss *et al*. 2003, Lam *et al*. 2011, Yager 2015), and induce chromosomal aberrations, thus confirming its properties as mutagenic and carcinogenic factor. Along the same lines, in luminal breast tumors, up-regulation of ER signal pathway has been associated with cell proliferation, cell survival, and therapy resistance (Yager & Davidson 2006, Pietras & Marquez-Garban 2007, Chang 2011). Although factors such as local synthesis of estrogen (Fabian *et al*. 2007), autocrine regulation of cell proliferation (Fabian *et al*. 2007, Tan *et al*. 2009), and cross talk with signaling from other growth factors have been associated with this up-regulation, the mechanisms underlying the action of ER are still not fully understood.

In summary, our results demonstrate that low doses of E_2_ and TAM may favor the production of specific chromosomal abnormalities in both ER+ and ER- breast cancer cells. This genotoxic effect is higher in those cell lines with *HER2* gene amplification. The induction of chromosomal alterations by E_2_ and TAM observed *in vitro* may support the contention that a careful assessment of the risk and the benefit of E_2_ and TAM administration should be considered. Indeed, the novel chromosomal rearrangements originated following E_2_ and TAM exposure may contribute to stimulate cell proliferation leading to survival advantages and allowing for selection of clones with new chromosomal abnormalities. *In vivo* studies that may help address the biological effect of such alterations and ascertain whether or not these may be responsible for treatment resistance are warranted.

## Supplementary data

This is linked to the online version of the paper at http://dx.doi.org/10.1530/ERC-16-0078.

## Declaration of interest

The authors declare that there is no conflict of interest that could be perceived as prejudicing the impartiality of the research reported.

## Funding

This work was funded by the Italian Association of Cancer Research, AIRC (MFAG13310 to C M), by the Ministry of University (Ex 60% 2014 and 2015 to C M) and by Fondazione Piemontese per la Ricerca sul Cancro (ONLUS) 5 X 1000 Fondi Ministero della Salute 2013 (to A S).

## Authors’ contribution statement

M R L performed the experiments and analyzed and interpreted the data. L V d C acquired and analyzed G-banding and M-FISH karyotypes. R R and L A participated in cell culture experiments. T M performed IHC. N R performed statistical analyses and participated in data analysis. I C participated in data analysis. C M and A S conceived and supervised the study and analyzed and interpreted the data. M R L, C M, and A S wrote the manuscript.

## References

[bib1] Achuthan R Bell SM Roberts P Leek JP Horgan K Markham AF MacLennan KA Speirs V 2001 Genetic events during the transformation of a tamoxifen-sensitive human breast cancer cell line into a drug-resistant clone. Cancer Genetics and Cytogenetics 130 166–172. (10.1016/S0165-4608(01)00475-7) 11675139

[bib2] Ahmad ME Shadab GG Hoda A Afzal M 2000 Genotoxic effects of estradiol-17beta on human lymphocyte chromosomes. Mutation Research 466 109–115. (10.1016/S1383-5718(99)00230-2) 10751732

[bib3] Al Sarraj J Vinson C Thiel G 2005 Regulation of asparagine synthetase gene transcription by the basic region leucine zipper transcription factors ATF5 and CHOP. Biological Chemistry 386 873–879. (10.1515/BC.2005.102) 16164412

[bib4] Azuma K Urano T Horie-Inoue K Hayashi S Sakai R Ouchi Y Inoue S 2009 Association of estrogen receptor alpha and histone deacetylase 6 causes rapid deacetylation of tubulin in breast cancer cells. Cancer Research 69 2935–2940. (10.1158/0008-5472.CAN-08-3458) 19318565

[bib5] Banham AH Beasley N Campo E Fernandez PL Fidler C Gatter K Jones M Mason DY Prime JE Trougouboff P 2001 The FOXP1 winged helix transcription factor is a novel candidate tumor suppressor gene on chromosome 3p. Cancer Research 61 8820–8829. 11751404

[bib6] Barrett JC Wong A McLachlan JA 1981 Diethylstilbestrol induces neoplastic transformation without measurable gene mutation at two loci. Science 212 1402–1404. (10.1126/science.6262919) 6262919

[bib7] Bernstein L Ross RK 1993 Endogenous hormones and breast cancer risk. Epidemiologic Reviews 15 48–65. 8405212 10.1093/oxfordjournals.epirev.a036116

[bib8] Beroukhim R Mermel CH Porter D Wei G Raychaudhuri S Donovan J Barretina J Boehm JS Dobson J Urashima M 2010 The landscape of somatic copy-number alteration across human cancers. Nature 463 899–905. (10.3410/f.2381956.2025054) 20164920 PMC2826709

[bib9] Berthois Y Katzenellenbogen JA Katzenellenbogen BS 1986 Phenol red in tissue culture media is a weak estrogen: implications concerning the study of estrogen-responsive cells in culture. PNAS 83 2496–2500. (10.1073/pnas.83.8.2496) 3458212 PMC323325

[bib10] Bignell GR Greenman CD Davies H Butler AP Edkins S Andrews JM Buck G Chen L Beare D Latimer C 2010 Signatures of mutation and selection in the cancer genome. Nature 463 893–898. (10.1038/nature08768) 20164919 PMC3145113

[bib11] Campiglio M Pekarsky Y Menard S Tagliabue E Pilotti S Croce CM 1999 FHIT loss of function in human primary breast cancer correlates with advanced stage of the disease. Cancer Research 59 3866–3869. 10463571

[bib12] Castellano-Diaz E Gonzalez-Quijano MI Liminana JM Diaz-Chico BN 1989 Tamoxifen decreases the estradiol induced progesterone receptors by interfering with nuclear estrogen receptor accumulation. Journal of Steroid Biochemistry 33 133–139. (10.1016/0022-4731(89)90368-3) 2761261

[bib13] Chang M 2011 Dual roles of estrogen metabolism in mammary carcinogenesis. BMB Reports 44 423–434. (10.5483/BMBRep.2011.44.7.423) 21777512

[bib14] Chelouche-Lev D Kluger HM Berger AJ Rimm DL Price JE 2004 alphaB-crystallin as a marker of lymph node involvement in breast carcinoma. Cancer 100 2543–2548. (10.1002/cncr.20304) 15197794

[bib15] Chen JQ Russo J 2009 ERalpha-negative and triple negative breast cancer: molecular features and potential therapeutic approaches. Biochimica et Biophysica Acta 1796 162–175. (10.1016/j.bbcan.2009.06.003) 19527773 PMC2937358

[bib16] Cheng SB Graeber CT Quinn JA Filardo EJ 2011 Retrograde transport of the transmembrane estrogen receptor, G-protein-coupled-receptor-30 (GPR30/GPER) from the plasma membrane towards the nucleus. Steroids 76 892–896. (10.1016/j.steroids.2011.02.018) 21354433

[bib17] Chetrite GS Cortes-Prieto J Philippe JC Wright F Pasqualini JR 2000 Comparison of estrogen concentrations, estrone sulfatase and aromatase activities in normal, and in cancerous, human breast tissues. Journal of Steroid Biochemistry and Molecular Biology 72 23–27. (10.1016/S0960-0760(00)00040-6) 10731634

[bib18] Cox DG Hankinson SE Hunter DJ 2006 Polymorphisms of the AURKA (STK15/Aurora Kinase) Gene and Breast Cancer Risk (United States). Cancer Causes and Control 17 81–83. (10.1007/s10552-005-0429-9) 16411056

[bib19] Cui X Schiff R Arpino G Osborne CK Lee AV 2005 Biology of progesterone receptor loss in breast cancer and its implications for endocrine therapy. Journal of Clinical Oncology 23 7721–7735. (10.1200/JCO.2005.09.004) 16234531

[bib20] Cui M Yu W Dong J Chen J Zhang X Liu Y 2010 Downregulation of ABI1 expression affects the progression and prognosis of human gastric carcinoma. Medical Oncology 27 632–639. (10.1007/s12032-009-9260-6) 19554484

[bib21] Curtis C Shah SP Chin SF Turashvili G Rueda OM Dunning MJ Speed D Lynch AG Samarajiwa S Yuan Y 2012 The genomic and transcriptomic architecture of 2,000 breast tumours reveals novel subgroups. Nature 486 346–352. (10.1038/nature10983) 22522925 PMC3440846

[bib22] Dayal J Albergant L Newman T South A 2015 Quantitation of multiclonality in control and drug-treated tumour populations using high-throughput analysis of karyotypic heterogeneity. Convergent Science Physical Oncology 1 025001 (10.1088/2057-1739/1/2/025001)

[bib23] Di Modugno F Mottolese M Di Benedetto A Conidi A Novelli F Perracchio L Venturo I Botti C Jager E Santoni A 2006 The cytoskeleton regulatory protein hMena (ENAH) is overexpressed in human benign breast lesions with high risk of transformation and human epidermal growth factor receptor-2-positive/hormonal receptor-negative tumors. Clinical Cancer Research 12 1470–1478. (10.1158/1078-0432.CCR-05-2027) 16533770

[bib24] Dossus L Kaaks R Canzian F Albanes D Berndt SI Boeing H Buring J Chanock SJ Clavel-Chapelon F Feigelson HS 2010 PTGS2 and IL6 genetic variation and risk of breast and prostate cancer: results from the Breast and Prostate Cancer Cohort Consortium (BPC3). Carcinogenesis 31 455–461. (10.1093/carcin/bgp307) 19965896 PMC2832545

[bib25] Dowsett M 1999 Drug and hormone interactions of aromatase inhibitors. Endocrine-Related Cancer 6 181–185. (10.1677/erc.0.0060181) 10731107

[bib26] Dowsett M Smith IE Ebbs SR Dixon JM Skene A A’Hern R Salter J Detre S Hills M Walsh G 2007 Prognostic value of Ki67 expression after short-term presurgical endocrine therapy for primary breast cancer. Journal of the National Cancer Institute 99 167–170. (10.1093/jnci/djk020) 17228000

[bib27] Early Breast Cancer Trialists’ Collaborative Group 1992 Systemic treatment of early breast cancer by hormonal, cytotoxic, or immune therapy. 133 randomised trials involving 31,000 recurrences and 24,000 deaths among 75,000 women. Lancet 339 71–85. (10.1016/0140-6736(92)90997-h) 1345869

[bib28] Early Breast Cancer Trialists’ Collaborative Group 1998 Tamoxifen for early breast cancer: an overview of the randomised trials. Lancet 351 1451–1467. (10.1016/s0140-6736(97)11423-4) 9605801

[bib29] Early Breast Cancer Trialists’ Collaborative Group, Davies C Godwin J Gray R Clarke M Cutter D Darby S McGale P Pan HC Taylor C 2011 Relevance of breast cancer hormone receptors and other factors to the efficacy of adjuvant tamoxifen: patient-level meta-analysis of randomised trials. Lancet 378 771–784. (10.1016/s0140-6736(11)60993-8) 21802721 PMC3163848

[bib30] Edwards AS Scott JD 2000 A-kinase anchoring proteins: protein kinase A and beyond. Current Opinion in Cell Biology 12 217–221. (10.1016/S0955-0674(99)00085-X) 10712918

[bib31] Fabian CJ Kimler BF Zalles CM Khan QJ Mayo MS Phillips TA Simonsen M Metheny T Petroff BK 2007 Reduction in proliferation with six months of letrozole in women on hormone replacement therapy. Breast Cancer Research and Treatment 106 75–84. (10.1007/s10549-006-9476-5) 17221152

[bib32] Gascoigne KE Cheeseman IM 2013 Induced dicentric chromosome formation promotes genomic rearrangements and tumorigenesis. Chromosome Research 21 407–418. (10.1007/s10577-013- 9368-6) 23793898 PMC3713265

[bib33] Greaves M Maley CC 2012 Clonal evolution in cancer. Nature 481 306–313. (10.1038/nature10762) 22258609 PMC3367003

[bib34] Gruvberger-Saal SK Bendahl PO Saal LH Laakso M Hegardt C Eden P Peterson C Malmstrom P Isola J Borg A 2007 Estrogen receptor beta expression is associated with tamoxifen response in ERalpha-negative breast carcinoma. Clinical Cancer Research 13 1987–1994. (10.1158/1078-0432.CCR-06-1823) 17404078

[bib35] Heng HH Liu G Bremer S Ye KJ Stevens J Ye CJ 2006 Clonal and non-clonal chromosome aberrations and genome variation and aberration. Genome 49 195–204. (10.1139/G06-023) 16604101

[bib36] Higashi E Fukami T Itoh M Kyo S Inoue M Yokoi T Nakajima M 2007 Human CYP2A6 is induced by estrogen via estrogen receptor. Drug Metabolism and Disposition 35 1935–1941. (10.1124/dmd.107.016568) 17646279

[bib37] Hu RJ Lee MP Connors TD Johnson LA Burn TC Su K Landes GM Feinberg AP 1997 A 2.5-Mb transcript map of a tumor-suppressing subchromosomal transferable fragment from 11p15.5, and isolation and sequence analysis of three novel genes. Genomics 46 9–17. (10.1006/geno.1997.4981) 9403053

[bib38] Hu P Kinyamu HK Wang L Martin J Archer TK Teng C 2008 Estrogen induces estrogen-related receptor alpha gene expression and chromatin structural changes in estrogen receptor (ER)-positive and ER-negative breast cancer cells. Journal of Biological Chemistry 283 6752–6763. (10.1074/jbc.M705937200) 18174157

[bib39] Igarashi T Izumi H Uchiumi T Nishio K Arao T Tanabe M Uramoto H Sugio K Yasumoto K Sasaguri Y 2007 Clock and ATF4 transcription system regulates drug resistance in human cancer cell lines. Oncogene 26 4749–4760. (10.1038/sj.onc.1210289) 17297441

[bib40] International Breast Cancer Study Group, Colleoni M Gelber S Goldhirsch A Aebi S Castiglione-Gertsch M Price KN Coates AS Gelber RD 2006 Tamoxifen after adjuvant chemotherapy for premenopausal women with lymph node-positive breast cancer: International Breast Cancer Study Group Trial 13-93. Journal of Clinical Oncology 24 1332–1341. (10.1200/jco.2005.03.0783) 16505417

[bib41] Ivanovic V Krtolica K Krajnovic M Dimitrijevic B 2006 Role of transforming growth factor-ß1 in breast carcinogenesis. Archive of Oncology 14 3 (10.2298/aoo0604122i)

[bib42] Ivyna Bong P Zubaidah Z Rohaizak M Naqiyah I Nor Aina E Noor Hisham A Sharifah N 2011 Elevated expression of CD151 gene in estrogen receptor and progesterone receptor positive breast carcinoma. Medicine and Health 6 7 (10.15208/mhsj)

[bib43] Jansen MP Foekens JA van Staveren IL Dirkzwager-Kiel MM Ritstier K Look MP Meijer-van Gelder ME Sieuwerts AM Portengen H Dorssers LC 2005 Molecular classification of tamoxifen-resistant breast carcinomas by gene expression profiling. Journal of Clinical Oncology 23 732–740. (10.1200/JCO.2005.05.145) 15681518

[bib44] Jeruss JS Liu NX Chung Y Magrane G Waldman F Edgerton S Yang X Thor AD 2003 Characterization and chromosomal instability of novel derived cell lines from a wt-erbB-2 transgenic mouse model. Carcinogenesis 24 659–664. (10.1093/carcin/bgg001) 12727793

[bib45] Johnston SR Dowsett M 2003 Aromatase inhibitors for breast cancer: lessons from the laboratory. Nature Reviews Cancer 3 821–831. (10.1038/nrc1211) 14668813

[bib46] Kamal M Shaaban AM Zhang L Walker C Gray S Thakker N Toomes C Speirs V Bell SM 2010 Loss of CSMD1 expression is associated with high tumour grade and poor survival in invasive ductal breast carcinoma. Breast Cancer Research and Treatment 121 555–563. (10.1007/s10549-009-0500-4) 19669408

[bib47] Kang HS Ahn SH Mishra SK Hong KM Lee ES Shin KH Ro J Lee KS Kim MK 2014 Association of polymorphisms and haplotypes in the insulin-like growth factor 1 receptor (IGF1R) gene with the risk of breast cancer in Korean women. PLoS ONE 9 e84532 (10.1371/journal.pone.0084532) 24392142 PMC3879335

[bib48] Kedia-Mokashi N Makawy AE Saxena M Balasinor NH 2010 Chromosomal aberration in the post-implantation embryos sired by tamoxifen treated male rats. Mutation Research 703 169–173. (10.1016/j.mrgentox.2010.08.016) 20801230

[bib49] Kim D Jung W Koo JS 2011 The expression of ERCC1, RRM1, and BRCA1 in breast cancer according to the immunohistochemical phenotypes. Journal of Korean Medical Science 26 352–359. (10.3346/jkms.2011.26.3.352) 21394302 PMC3051081

[bib50] Koren R Dekel Y Sherman E Weissman Y Dreznik Z Klein B Gal R 2003 The expression of DCC protein in female breast cancer. Breast Cancer Research and Treatment 80 215–220. (10.1023/A:1024581508474) 12908825

[bib51] Kousidou OC Roussidis AE Theocharis AD Karamanos NK 2004 Expression of MMPs and TIMPs genes in human breast cancer epithelial cells depends on cell culture conditions and is associated with their invasive potential. Anticancer Research 24 4025–4030. 15739263

[bib52] Kumar R Zakharov MN Khan SH Miki R Jang H Toraldo G Singh R Bhasin S Jasuja R 2011 The dynamic structure of the estrogen receptor. Journal of Amino Acids 2011 812540 (10.4061/2011/812540) 22312471 PMC3268042

[bib53] Lam SH Lee SG Lin CY Thomsen JS Fu PY Murthy KR Li H Govindarajan KR Nick LC Bourque G 2011 Molecular conservation of estrogen-response associated with cell cycle regulation, hormonal carcinogenesis and cancer in zebrafish and human cancer cell lines. BMC Medical Genomics 4 41 (10.1186/1755-8794-4-41) 21575170 PMC3114699

[bib54] Laskowska E Kuczynska-Wisnik D Matuszewska E 2010 HSPB1 (Heat-Shock 27 kDa Protein 1). Atlas of Genetics and Cytogenetics in Oncology and Haematology 14 130–136. (10.4267/ 2042/44685)

[bib55] Lazzeroni M Serrano D Dunn BK Heckman-Stoddard BM Lee O Khan S Decensi A 2012 Oral low dose and topical tamoxifen for breast cancer prevention: modern approaches for an old drug. Breast Cancer Research 14 214 (10.1186/bcr3233) 23106852 PMC4053098

[bib56] Lee ST Lee JY Han CR Kim YH Jun do Y Taub D Kim YH 2015 Dependency of 2-methoxyestradiol-induced mitochondrial apoptosis on mitotic spindle network impairment and prometaphase arrest in human Jurkat T cells. Biochemical Pharmacology 94 257–269. (10.1016/j.bcp.2015.02.011) 25732194

[bib57] Li JJ Weroha SJ Lingle WL Papa D Salisbury JL Li SA 2004 Estrogen mediates Aurora-A overexpression, centrosome amplification, chromosomal instability, and breast cancer in female ACI rats. PNAS 101 18123–18128. (10.1073/pnas.0408273101) 15601761 PMC539804

[bib58] Li Y Birnbaumer L Teng CT 2010 Regulation of ERRalpha gene expression by estrogen receptor agonists and antagonists in SKBR3 breast cancer cells: differential molecular mechanisms mediated by g protein-coupled receptor GPR30/GPER-1. Molecular Endocrinology 24 969–980. (10.1210/me.2009-0148) 20211987 PMC2870941

[bib59] Liehr JG 2000 Is estradiol a genotoxic mutagenic carcinogen? Endocrine Reviews 21 40–54. (10.1210/er.21.1.40) 10696569

[bib60] Lin P 2009 BCL10 (B-cell CLL/lymphoma 10). Atlas of Genetics and Cytogenetics in Oncology and Haematology 12 1 (10.4267/2042/44630)

[bib61] Lincoln DW 2nd Bove K 2005 The transcription factor Ets-1 in breast cancer. Frontiers in Bioscience 10 506–511. (10.2741/1546) 15574387

[bib62] Liu G Stevens JB Horne SD Abdallah BY Ye KJ Bremer SW Ye CJ Chen DJ Heng HH 2014 Genome chaos: survival strategy during crisis. Cell Cycle 13 528–537. (10.4161/cc.27378) 24299711 PMC6093293

[bib63] Ljuslinder I Malmer B Golovleva I Thomasson M Grankvist K Hockenstrom T Emdin S Jonsson Y Hedman H Henriksson R 2005 Increased copy number at 3p14 in breast cancer. Breast Cancer Research 7 R719–R727. (10.1186/bcr1279) 16168117 PMC1242137

[bib64] Lundgren K Holm K Nordenskjold B Borg A Landberg G 2008 Gene products of chromosome 11q and their association with CCND1 gene amplification and tamoxifen resistance in premenopausal breast cancer. Breast Cancer Research 10 R81 (10.1186/bcr2150) 18823530 PMC2614516

[bib65] Luo W Wood CG Earley K Hung MC Lin SH 1997 Suppression of tumorigenicity of breast cancer cells by an epithelial cell adhesion molecule (C-CAM1): the adhesion and growth suppression are mediated by different domains. Oncogene 14 7 (10.1038/sj.onc.1200999) 9135071

[bib66] McGuire WL 1975 Current status of estrogen receptors in human breast cancer. Cancer 36 638–644. (10.1002/1097-0142(197508)36:2+<638::aid-cncr2820360805>3.0.co;2-s) 168960

[bib67] Mizutani A Okada T Shibutani S Sonoda E Hochegger H Nishigori C Miyachi Y Takeda S Yamazoe M 2004 Extensive chromosomal breaks are induced by tamoxifen and estrogen in DNA repair-deficient cells. Cancer Research 64 3144–3147. (10.1158/0008-5472.CAN-03-3489) 15126352

[bib68] Nakatani K Thompson DA Barthel A Sakaue H Liu W Weigel RJ Roth RA 1999 Up-regulation of Akt3 in estrogen receptor-deficient breast cancers and androgen-independent prostate cancer lines. Journal of Biological Chemistry 274 21528–21532. (10.1074/jbc.274.31.21528) 10419456

[bib69] Nik-Zainal S Alexandrov LB Wedge DC Van Loo P Greenman CD Raine K Jones D Hinton J Marshall J Stebbings LA 2012 Mutational processes molding the genomes of 21 breast cancers. Cell 149 979–993. (10.1016/j.cell.2012.04.024) 22608084 PMC3414841

[bib70] O’Brien SL Fagan A Fox EJ Millikan RC Culhane AC Brennan DJ McCann AH Hegarty S Moyna S Duffy MJ 2007 CENP-F expression is associated with poor prognosis and chromosomal instability in patients with primary breast cancer. International Journal of Cancer 120 1434–1443. (10.1002/ijc.22413) 17205517 PMC4972098

[bib71] Orsetti B Nugoli M Cervera N Lasorsa L Chuchana P Rouge C Ursule L Nguyen C Bibeau F Rodriguez C 2006 Genetic profiling of chromosome 1 in breast cancer: mapping of regions of gains and losses and identification of candidate genes on 1q. British Journal of Cancer 95 1439–1447. (10.1038/sj.bjc.6603433) 17060936 PMC2360604

[bib72] Pearce ST Jordan VC 2004 The biological role of estrogen receptors alpha and beta in cancer. Critical Reviews in Oncology/Hematology 50 3–22. (10.1016/j.critrevonc.2003.09.003) 15094156

[bib73] Pierantoni GM Rinaldo C Mottolese M Di Benedetto A Esposito F Soddu S Fusco A 2007 High-mobility group A1 inhibits p53 by cytoplasmic relocalization of its proapoptotic activator HIPK2. Journal of Clinical Investigation 117 693–702. (10.1172/JCI29852) 17290307 PMC1784001

[bib74] Pietras RJ Marquez-Garban DC 2007 Membrane-associated estrogen receptor signaling pathways in human cancers. Clinical Cancer Research 13 4672–4676. (10.1158/1078-0432.CCR-07-1373) 17699844

[bib75] Popescu NC Zimonjic DB 2002 Chromosome and gene alterations in breast cancer as markers for diagnosis and prognosis as well as pathogenetic targets for therapy. American Journal of Medical Genetics 115 142–149. (10.1002/ajmg.10696) 12407694

[bib76] Quick EL Parry EM Parry JM 2008 Do oestrogens induce chromosome specific aneuploidy in vitro, similar to the pattern of aneuploidy seen in breast cancer? Mutation Research 651 46–55. (10.1016/j.mrgentox.2007.10.021) 18162433

[bib77] Rabbitts TH 1994 Chromosomal translocations in human cancer. Nature 372 143–149. (10.1038/372143a0) 7969446

[bib78] Radestock Y Hoang-Vu C Hombach-Klonisch S 2008 Relaxin reduces xenograft tumour growth of human MDA-MB-231 breast cancer cells. Breast Cancer Research 10 R71 (10.1186/ bcr2136) 18718015 PMC2575545

[bib79] Rafn B Nielsen CF Andersen SH Szyniarowski P Corcelle-Termeau E Valo E Fehrenbacher N Olsen CJ Daugaard M Egebjerg C 2012 ErbB2-driven breast cancer cell invasion depends on a complex signaling network activating myeloid zinc finger-1-dependent cathepsin B expression. Molecular Cell 45 764–776. (10.1016/j.molcel.2012.01.029) 22464443

[bib80] Riethdorf L Lisboa BW Henkel U Naumann M Wagener C Loning T 1997 Differential expression of CD66a (BGP), a cell adhesion molecule of the carcinoembryonic antigen family, in benign, premalignant, and malignant lesions of the human mammary gland. Journal of Histochemistry and Cytochemistry 45 957–963. (10.1177/002215549704500705) 9212821

[bib81] Rodrigues-Ferreira S Di Tommaso A Dimitrov A Cazaubon S Gruel N Colasson H Nicolas A Chaverot N Molinie V Reyal F 2009 8p22 MTUS1 gene product ATIP3 is a novel anti-mitotic protein underexpressed in invasive breast carcinoma of poor prognosis. PLoS ONE 4 e7239 (10.1371/journal.pone.0007239) 19794912 PMC2749209

[bib82] Rondon-Lagos M Verdun Di Cantogno L Marchio C Rangel N Payan-Gomez C Gugliotta P Botta C Bussolati G Ramirez-Clavijo SR Pasini B 2014a Differences and homologies of chromosomal alterations within and between breast cancer cell lines: a clustering analysis. Molecular Cytogenetics 7 8 (10.1186/1755-8166-7-8) 24456987 PMC3914704

[bib83] Rondon-Lagos M Verdun Di Cantogno L Rangel N Mele T Ramirez-Clavijo SR Scagliotti G Marchio C Sapino A 2014b Unraveling the chromosome 17 patterns of FISH in interphase nuclei: an in-depth analysis of the HER2 amplicon and chromosome 17 centromere by karyotyping, FISH and M-FISH in breast cancer cells. BMC Cancer 14 922 (10.1186/1471-2407-14-922) 25481507 PMC4295336

[bib84] Roy D Calaf GM Hande MP Hei TK 2006 Allelic imbalance at 11q23-q24 chromosome associated with estrogen and radiation-induced breast cancer progression. International Journal of Oncology 28 667–674. (10.3892/ijo.28.3.667) 16465372

[bib85] Russo J Russo IH 2006 The role of estrogen in the initiation of breast cancer. Journal of Steroid Biochemistry and Molecular Biology 102 89–96. (10.1016/j.jsbmb.2006.09.004) 17113977 PMC1832080

[bib86] Sapino A Pietribiasi F Bussolati G Marchisio PC 1986 Estrogen- and tamoxifen-induced rearrangement of cytoskeletal and adhesion structures in breast cancer MCF-7 cells. Cancer Research 46 2526–2531. 2421880

[bib87] Sato Y Sakakibara Y Oda T Aizu-Yokota E Ichinoseki K 1992 Effect of estradiol and ethynylestradiol on microtubule distribution in Chinese hamster V79 cells. Chemical and Pharmaceutical Bulletin 40 182–184. (10.1248/cpb.40.182) 1576671

[bib88] Sattler M Quinnan LR Pride YB Gramlich JL Chu SC Even GC Kraeft SK Chen LB Salgia R 2003 2-methoxyestradiol alters cell motility, migration, and adhesion. Blood 102 289–296. (10.1182/blood-2002-03-0729) 12637335

[bib89] Scibetta AG Wong PP Chan KV Canosa M Hurst HC 2010 Dual association by TFAP2A during activation of the p21cip/CDKN1A promoter. Cell Cycle 9 4525–4532. (10.4161/cc.9.22.13746) 21084835 PMC3048049

[bib90] Scintu M Vitale R Prencipe M Gallo AP Bonghi L Valori VM Maiello E Rinaldi M Signori E Rabitti C 2007 Genomic instability and increased expression of BUB1B and MAD2L1 genes in ductal breast carcinoma. Cancer Letters 254 298–307. (10.1016/j.canlet.2007.03.021) 17498870

[bib91] Shaffer LG McGowan-Jordan J Schmid M 2013 ISCN: An International System for Human Cytogenetic Nomenclature. Basel, Switzerland: S. Karger.

[bib92] Shen MM 2013 Chromoplexy: a new category of complex rearrangements in the cancer genome. Cancer Cell 23 567–569. (10.1016/j.ccr.2013.04.025) 23680143 PMC3673705

[bib93] Shou J Massarweh S Osborne CK Wakeling AE Ali S Weiss H Schiff R 2004 Mechanisms of tamoxifen resistance: increased estrogen receptor-HER2/neu cross-talk in ER/HER2-positive breast cancer. Journal of the National Cancer Institute 96 926–935. (10.1093/jnci/djh166) 15199112

[bib94] Sreeramaneni R Chaudhry A McMahon M Sherr CJ Inoue K 2005 Ras-Raf-Arf signaling critically depends on the Dmp1 transcription factor. Molecular and Cellular Biology 25 220–232. (10.1128/MCB.25.1.220-232.2005) 15601844 PMC538777

[bib95] Stender JD Frasor J Komm B Chang KC Kraus WL Katzenellenbogen BS 2007 Estrogen-regulated gene networks in human breast cancer cells: involvement of E2F1 in the regulation of cell proliferation. Molecular Endocrinology 21 2112–2123. (10.1210/me.2006-0474) 17550982

[bib96] Tan H Zhong Y Pan Z 2009 Autocrine regulation of cell proliferation by estrogen receptor-alpha in estrogen receptor-alpha-positive breast cancer cell lines. BMC Cancer 9 31 (10.1186/1471-2407-9-31) 19171042 PMC2636826

[bib97] Thomas P Pang Y Filardo EJ Dong J 2005 Identity of an estrogen membrane receptor coupled to a G protein in human breast cancer cells. Endocrinology 146 624–632. (10.1210/en.2004-1064) 15539556

[bib98] Tsutsui T Barrett JC 1997 Neoplastic transformation of cultured mammalian cells by estrogens and estrogen like chemicals. Environmental Health Perspectives 105 (Supplement 3) 619–624. (10.1289/ehp.97105s3619) 9168005 PMC1469887

[bib99] Wang D Hu L Zhang G Zhang L Chen C 2010 G protein-coupled receptor 30 in tumor development. Endocrine 38 29–37. (10.1007/s12020-010-9363-z) 20960099

[bib100] Watatani Y Kimura N Shimizu YI Akiyama I Tonaki D Hirose H Takahashi S Takahashi Y 2007 Amino acid limitation induces expression of ATF5 mRNA at the post-transcriptional level. Life Sciences 80 879–885. (10.1016/j.lfs.2006.11.013) 17140605

[bib101] Yager JD 2015 Mechanisms of estrogen carcinogenesis: the role of E2/E1-quinone metabolites suggests new approaches to preventive intervention a review. Steroids 99 56–60. (10.1016/j.steroids.2014.08.006) 25159108 PMC4339663

[bib102] Yager JD Davidson NE 2006 Estrogen carcinogenesis in breast cancer. New England Journal of Medicine 354 270–282. (10.1056/NEJMra050776) 16421368

[bib103] Yong HY Hwang JS Son H Park HI Oh ES Kim HH Kim DK Choi WS Lee BJ Kim HR 2011 Identification of H-Ras-specific motif for the activation of invasive signaling program in human breast epithelial cells. Neoplasia 13 8 (10.1593/neo.101088) PMC3033589 21403836

[bib104] Zaretsky JZ Barnea I Aylon Y Gorivodsky M Wreschner DH Keydar I 2006 MUC1 gene overexpressed in breast cancer: structure and transcriptional activity of the MUC1 promoter and role of estrogen receptor alpha (ERalpha) in regulation of the MUC1 gene expression. Molecular Cancer 5 57 (10.1186/1476-4598-5-57) 17083744 PMC1636664

[bib105] Zhang CZ Leibowitz ML Pellman D 2013 Chromothripsis and beyond: rapid genome evolution from complex chromosomal rearrangements. Genes and Development 27 2513–2530. (10.1101/gad.229559.113) 24298051 PMC3861665

